# A study of association between common variation in the growth hormone-chorionic somatomammotropin hormone gene cluster and adult fasting insulin in a UK Caucasian population

**DOI:** 10.1186/1477-5751-5-18

**Published:** 2006-11-24

**Authors:** Rachel M Freathy, Simon MS Mitchell, Beatrice Knight, Beverley Shields, Michael N Weedon, Andrew T Hattersley, Timothy M Frayling

**Affiliations:** 1 Institute of Biomedical and Clinical Science, Peninsula Medical School, Exeter, UK

## Abstract

**Background:**

Reduced growth during infancy is associated with adult insulin resistance. In a UK Caucasian cohort, the *CSH1.01 *microsatellite polymorphism in the growth hormone-chorionic somatomammotropin hormone gene cluster was recently associated with increases in adult fasting insulin of approximately 23 pmol/l for TT homozygote males compared to D1D1 or D2D2 homozygotes (*P *= 0.001 and 0.009; *n *= 206 and 92, respectively), but not for females. TT males additionally had a 547-g lower weight at 1 year (*n *= 270; *P = *0.008) than D2D2 males. We sought to replicate these data in healthy UK Caucasian subjects. We genotyped 1396 subjects (fathers, mothers and children) from a consecutive birth study for the *CSH1.01 *marker and analysed genotypes for association with 1-year weight in boys and fasting insulin in fathers.

**Results:**

We found no evidence for association of *CSH1.01 *genotype with adult male fasting insulin concentrations (TT/D1D1 *P = *0.38; TT/D2D2 *P *= 0.18) or weight at 1 year in boys (TT/D1D1 *P = *0.76; TT/D2D2 *P *= 0.85). For fasting insulin, our data can exclude the previously observed effect sizes as the 95 % confidence intervals for the differences observed in our study exclude increases in fasting insulin of 9.0 and 12.6 pmol/l for TT relative to D1D1 and D2D2 homozygotes, respectively. Whilst we have fewer data on boys' 1-year weight than the original study, our data can exclude a reduction in 1-year weight greater than 557 g for TT relative to D2D2 homozygotes.

**Conclusion:**

We have not found association of the *CSH1.01 *genotype with fasting insulin or weight at 1 year. We conclude that the original study is likely to have over-estimated the effect size for fasting insulin, or that the difference in results reflects the younger age of subjects in this study relative to those in the previous study.

## Background

Reduced birth weight and reduced growth in infancy are associated with adult disorders characterised by insulin resistance in the general population [1-5]. These include type 2 diabetes, coronary heart disease and hypertension. Their association with birth weight may be explained by programming of metabolism due to undernutrition *in utero *[1], or by genetic factors: common genetic variants which increase insulin resistance may predispose both to low insulin-mediated growth *in utero *and insulin resistance in adulthood [6]. It has been proposed that infant length or weight measured up to the age of two increasingly reflects the influence of the infant's own genes on the growth trajectory since the influence of the maternal intra-uterine environment is no longer present [7,8]. Since reduced weight in infancy is also associated with adult insulin resistance, candidate genes with effects on both of these traits, as well as birth weight may explain the observed associations.

Few genes are known to influence both diabetes-related traits and birth weight. Positive associations with both phenotypes have been shown for the insulin gene *(INS*) variable number of tandem repeats (VNTR) locus [8-10], a microsatellite polymorphism in the insulin-like growth factor 1 gene (*IGF1*) [11,12] and the glucokinase gene *GCK*(-30) polymorphism [13]. There is evidence that a single nucleotide polymorphism in complete linkage disequilibrium with *INS-*VNTR classes I and III (rs689) is functional [14]. Despite this, studies attempting to replicate the *INS*-VNTR and *IGF1 *associations have produced inconsistent results [15-20]. Replication of any genetic association study is vital for determining whether the observed association is real, since it increases the cumulative sample size and helps to guard against the low *a priori *odds of a variant altering a phenotype, which may hinder any single study [21,22].

Recently, Day et al. [23] reported that genetic variation in the *GH/CSH *gene cluster, which includes growth hormone (*GH1*; chromosome 17q23), is associated with altered 1-year weight and adult insulin resistance in UK Caucasian males aged 59–72 years. Variation in a highly polymorphic microsatellite marker, *CSH1.01*, was dichotomised into allele lineages based on possession of a dinucleotide repeat allele (D1; D2 (subset)) or a tetranucleotide repeat allele (T). In male subjects from north and east Hertfordshire, TT homozygotes had a 64.6 % (22.8 pmol/l) or 66.5 % (23.2 pmol/l) higher fasting insulin compared to D1D1 homozygotes (*P *= 0.001; *n *= 206) and D2D2 homozygotes (*P *= 0.009; *n *= 92), respectively. The TT genotype was also associated with a 5.3 % (547 g) reduction in weight at 1 year compared to the D2D2 genotype (*P = *0.008; *n *= 270) but this difference was not observed when compared to the D1D1 genotype (*P *= 0.24; *n *= 593). There was no association of genotype with birth weight, and no association with any measured phenotype in females.

Strong linkage disequilibrium occurs across the 66.5-kb *GH/CSH *gene cluster such that variation in two or more of the genes, inherited together, may reduce growth in early life while predisposing to disease later in life [23]. The gene cluster is an excellent candidate region for predisposing to restricted early growth and later insulin resistance. Placental growth hormone (*GH2*) and chorionic somatomammotropin (human placental lactogen) hormones 1 and 2 (*CSH1 *and *CSH2*), are expressed in the placenta and are involved in regulating fetal glucose supply and growth [24,25]. *GH1*, through transcriptional regulation of the gene for insulin-like growth factor-I (*IGF1*) and related genes, has a critical role in the regulation of postnatal growth [26]. Exogenous growth hormone administration alters glucose metabolism both *in vitro *and *in vivo *[27], whilst growth hormone deficiency and acromegaly are characterised respectively by sensitivity and resistance to insulin [28]. In addition, lower circulating IGF-I concentrations are associated with higher risk of impaired glucose tolerance or type 2 diabetes [29].

We sought replication of the associations reported by Day et al. [23]. We used healthy subjects (483 fathers, 479 mothers and 434 children) from a population-based consecutive birth study to assess the role of *CSH1.01 *variation in measures of fetal and postnatal growth and adult insulin resistance, as measured by fasting insulin concentrations and Homeostasis Model Assessment of Insulin Sensitivity (HOMA %S).

## Results

### *CSH1.01* genotype and fathers' fasting insulin

There was no association between father's D1/T or D2/T genotype and fasting insulin (Table 1). The *P *values for fathers' fasting insulin were little changed by adjustment for age and BMI (*P = *0.53 and 0.29 for the D1/T and D2/T genotypes, respectively).

**Table 1:** Phenotypes previously reported as associated with the *CSH1.01 *microsatellite by Day *et al*. [23]: Fathers' fasting insulin and boys' 1 year weight (with 95% confidence limits) tabulated for *CSH1.01 *allele group D1/T and D2/T genotypes

	T/T	D1/T	D1/D1	N	*P*	T/T	D2/T	D2/D2	N	*P*
**Fathers: fasting insulin (pmol/l)**	54.1 (46.1–63.5)	51.6 (47.9–55.7)	55.8 (51.4–60.7)	472	0.375	54.1 (45.9–63.7)	48.1 (42.9–54.0)	57.9 (48.3–69.3)	196	0.183
**Children: 1 yr weight (g)**	9786 (9457–10115)	9992 (9818–10167)	9946 (9763–10129)	366	0.554	9786 (9461–10112)	9914 (9670–10157)	10057 (9669–10445)	164	0.572
**Girls: 1 yr weight (g)**	9087 (8638–9536)	9610 (9375–9846)	9633 (9388–9877)	176	0.092	9087 (8648–9526)	9541 (9216–9867)	9688 (9117–10259)	75	0.164
**Boys: 1 yr weight (g)**	10401 (9969–10834)	10344 (10112–10575)	10242 (9998–10487)	190	0.758	10401 (9984–10819)	10252 (9937–10567)	10297 (9829–10764)	89	0.851

Fasting insulin and weight measures are unadjusted mean (95 % confidence limits). Fasting insulin values were log-transformed before analysis. *P *values are for one-way ANOVA.

### *CSH1.01* genotype and children's weight at 1 year

There was no association between children's D1/T or D2/T genotype and weight at 1 year (Table 1 shows results for all children, and also separately for boys and girls). The *P *values for children's 1 yr weight were little changed by adjustment for sex (*P = *0.49 and 0.66 for the D1/T and D2/T genotypes, respectively).

### *CSH1.01* genotype and other relevant phenotypes

There was also no association of D1/T or D2/T genotype with fathers' HOMA %S, children's birth weight (all children born at 36 weeks gestation or more, or stratified by sex) or placental weight (gestation 36 weeks or more), father or mother's height, father or mother's birth weight (obtained from subjects' mothers), father's BMI, mother's pre-pregnancy BMI, mother's age, father's age, father's fasting triglyceride concentrations adult or children's sex (all *P *> 0.05; results not shown). Oral glucose tolerance test and blood pressure data were not available for these subjects.

## Discussion

Common variants in the *GH-CSH *cluster are excellent candidates for contributing to common variation in fetal/infant growth and adult insulin resistance. The placentally-expressed *CSH1*, *CSH2 *and *GH2 *genes have key roles in the regulation of fetal glucose supply and growth [24,25], while *GH1 *has a critical role in postnatal growth and glucose metabolism [26-29]. A previous study reported that a microsatellite polymorphism in this cluster, *CSH1.01*, was associated with reduced weight at 1 year and increased fasting insulin concentrations in adult UK Caucasian males [23]. We have examined this polymorphism in an independent UK Caucasian study and found no evidence of association with either phenotype.

Our study included over twice as many adult male subjects for the fasting insulin analysis as the previous study [23]. This gave us more statistical power to detect an effect of genotype. Whereas Day et al. [23] showed that fasting insulin concentrations of TT carriers were 22.8 pmol/l higher than those of D1D1 carriers and 23.2 pmol/l higher than those of D2D2 carriers (*P = *0.009 and 0.008, respectively), we found no evidence of a difference and the 95 % confidence limits for the differences observed in our study exclude increases in fasting insulin above 9.0 and 12.6 pmol/l for TT relative to D1D1 and D2D2 homozygotes respectively. Using unlogged fasting insulin data, we obtained a more conservative estimate, but still are able to exclude increases in fasting insulin above 15.4 and 17.8 pmol/l for TT relative to D1D1 and D2D2 homozygotes, respectively. Our data suggest that the initial finding may have been a false-positive result or an over-estimation of the effect size. Both of these are a common problem for genetic association studies [30]. Further large-scale studies involving thousands, or tens of thousands of subjects will be required to investigate the possibility of smaller effect sizes. Another factor which may account for the differing result is that adult males in our study were younger (median age 33 years) than those in the previous study (age range 59–72 years). It is possible that the relationship between genotype and fasting insulin is modified by age. Some studies have reported gene-age interactions after results across all ages showed a weak association, for example the recent study of the relationship between the Leu262Val variant in the *PSARL *gene and plasma insulin in human subjects [31]. As with simple gene-phenotype associations, these interactions require replication. To investigate this possibility further, it will be necessary to carry out large-scale studies of *CSH1.01 *and fasting insulin in individuals spanning a wide range of ages.

We found no evidence of an association of *CSH1.01 *genotype with weight at 1 year. This contrasts with the results of Day et al. [23], who reported a 547 g reduction in weight at 1 year (*P = *0.008; *n *= 270) for TT compared to D2D2 males. Our data show a non-significant trend of 1-year weight values across the D1/T genotypes, in the opposite direction to that observed in the original study. Whilst we had reduced power to detect differences in weight at 1 year, the 95 % confidence limits for the difference observed in our study (-557 g, +767 g) exclude reductions in 1-year weight greater than 557 g for TT relative to D2D2 homozygote males. Whilst our data on female weight at 1 year are suggestive of an association of the same magnitude and direction as was seen for D2/T males in the original study, we acknowledge our reduced statistical power and conclude that further well-powered studies will be needed to confirm the role of this variant in fetal and infant growth.

This study focused on one variant within the *GH-CSH *gene cluster. Whilst we have not captured fully the common variation in this candidate region, we have examined a polymorphism previously associated with fasting insulin in males with a large effect size, but found no evidence for this in our larger sample.

## Conclusion

Replication of genetic association data in independent studies is vital for determining whether an initially observed association is a consistent finding. We have found no evidence that the *CSH1.01 *microsatellite polymorphism is associated with adult male fasting insulin in this larger replication study. We conclude that the result of the initial association study [23] is either a false positive, an over-estimation of the effect size for this phenotype, or a reflection of substantial heterogeneity between the two samples as a result of age differences. Further large-scale studies which capture more of the variation in the *GH-CSH *region will clarify its potential role in influencing fetal and infant growth and adult insulin resistance.

## Methods

### Subjects

Subjects were UK Caucasian fathers (*n *= 483), mothers (*n *= 479) and children (*n *= 434) from the Exeter Family Study of Childhood Health [32]. The clinical characteristics of subjects are shown in Table 2. All recruited subjects gave their informed consent. The study was approved by local research ethics committee and the protocol conforms to the ethical guidelines of the World Medical Association Declaration of Helsinki.

**Table 2:** Clinical characteristics of subjects

	Exeter Family Study Subjects		
	Fathers	Mothers	Children

*n*	483	479	434
Male (%)	-	-	51.6
Birth weight for children born >36 weeks gestation (g)	-	-	3500 (3175–3780)
Weight at 1 year (g)	-	-	9854 (9146–10730)^‡^
Age (years)	33 (29–36)	31 (27–34)	-
BMI (kg/m^2^)	26.2 (24.0–28.7)	23.1 (21.2–25.4)*	-
Height (cm)	178.0 (173.5–182.4)	164.9 (160.7–169.2)	-
Reported birth weight (g)	3402 (3005–3770)	3289 (3005–3629)	-
Fasting blood glucose (mmol/l)	4.7 (4.4–5.0)	4.3 (4.1–4.6)^†^	-
Fasting insulin (pmol/l)	49.0 (37.0–78.1)	59.2 (43.4–85.0)^†^	-

Continuous data are given as median (interquartile range). Subjects successfully genotyped for the CSH1.01 microsatellite are included.

*Pre-pregnancy BMI.

^†^Measurements taken at 28 weeks gestation.

^‡^Data on weight at 1 year were available for 366 children.

### Genotyping and quality control

Genomic DNA was isolated from leukocytes using standard techniques. The *CSH1.01 *microsatellite polymorphism was amplified by PCR using the following primers: forward 5'-GTT TAC TGC ACT CCA GCC TCG GAG-3'; reverse 5'-ACA AAA GTC CTT TCT CCA GAG CA-3'. A 5'-GTTT "PIGtail" was added to the forward primer to reduce the occurrence of non-templated A-addition. The forward primer was also labelled with the FAM fluorochrome. The PCR was performed in a final volume of 10 μl containing 16 ng genomic DNA, 2.5 pmol each primer, 2.25 mmol/l MgCl_2_, 0.25 mmol/l each of deoxy-ATP, -CTP, -GTP and -TTP and 0.25 units Amplitaq Gold DNA polymerase (Applied Biosystems, Warrington, UK). The reaction started with 12 min denaturation at 94°C, followed by 12 cycles of denaturation at 94°C for 30 s, annealing at 54°C for 30 s and extension at 72°C for 1 min. For 23 more cycles, the denaturation temperature was lowered to 89°C. The PCR was completed by a final extension at 72°C for 10 min. Products were separated on a standard polyacrylamide sequencing gel using the ABI377 autosequencer and analysed using GENESCAN and GENOTYPER software (Applied Biosystems). Control samples of known genotype were used in each PCR and every polyacrylamide gel to monitor genotyping consistency. These initially included samples genotyped in the previous study [23] for comparison. Negative controls (H_2_O) were also included to monitor potential contamination. The overall genotyping assay success rate was 83 %. Genotypes were in Hardy-Weinberg Equilibrium (*P *= 0.98 and 0.66 for D1/T and D2/T genotypes respectively). Genotyping accuracy, as determined from the genotype concordance between duplicate samples (11 % of total) was 99 %. Families showing Mendelian inconsistencies were excluded from analyses. Allele frequency distributions were similar in our study to that of Day et al. with a T allele frequency of 0.35, similar to the previously-reported figure of 0.34 [23].

### Classification of *CSH1.01* alleles and statistical analyses

**Figure 1:**
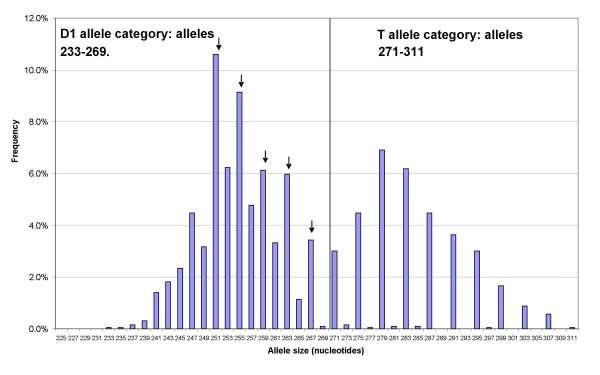
***CSH1.01 *allele frequency distribution (Exeter Family Study parents; N = 1924 alleles)**. Alleles marked by arrows were excluded from the D1 allele category to form the D2 category.

Alleles were dichotomized into D1/T or D2/T allele categories in exactly the same way as for the study by Day *et al*. [23]: alleles 271–311 nt were classified as T; alleles less than 271 nt were classified as D1; alleles 251, 255, 259, 263 and 267 nt were excluded from the D1 group to define the D2 group. Decisions relating to placement of the D1/T boundary and exclusion of alleles to create the D2 allele group were informed by comparison of our allele frequency distribution with that reported by Day *et al*. [23]. The *CSH1.01 *allele frequency distribution is shown in Figure 1.

We used Chi-squared tests to assess whether the genotypes of parents were in Hardy-Weinberg Equilibrium. We used General Linear Models in SPSS v.11.5 for Windows to test for association between D1/T or D2/T genotype and selected phenotypes of fathers, mothers or children: fasting insulin and HOMA %S (fathers only: mothers were pregnant (28 weeks gestation) at the time of data collection); height (mothers and fathers); placental weight; 1-year weight and birth weight (children). Analyses were performed both on uncorrected data and on data corrected for age and BMI (fasting insulin), sex and gestation (birth weight; placental weight) and sex (1 year weight). The 95 % confidence limits for the differences in fasting insulin observed in our study (TT relative to D1D1 and D2D2 homozygotes) were calculated using the antilog transformation and converting from the ratios obtained [33]

We had > 92 % power to detect the differences in adult male fasting insulin observed in the previously published study, i.e. increases of 22.8 pmol/l for TT relative to D1D1 homozygotes, and 23.2 pmol/l for TT relative to D2D2 homozygotes [23]. We had 80 % power to detect increases in adult male fasting insulin of 13.6 pmol/l for TT relative to D1D1 homozygotes, and of 18.9 pmol/l for TT relative to D2D2 homozygotes (*P *< 0.05 for difference in same direction as original study, assuming T allele frequency of 0.35). We had 80 % power to detect decreases in boys' weight at 1 year of 830 g for TT relative to D2D2 homozygotes (*P *value < 0.05; same direction as original study). We had reduced power (50 %) to detect the decrease of 547 g originally observed [23].

## Abbreviations

BMI, body mass index; *CSH1*, chorionic somatomammotropin hormone 1; *CSH2*, chorionic somatomammotropin hormone 2; *GH1*, growth hormone; *GH2*, placental growth hormone; *GH-CSH*, growth hormone-chorionic somatomammotropin hormone gene cluster; HOMA %S, Homeostasis Model Assessment of Insulin Sensitivity; *IGF1*, insulin-like growth factor 1; *INS-*VNTR, insulin gene variable number of tandem repeats; PCR, polymerase chain reaction.

## Competing interests

The authors declare that they have no competing interests.

## Authors' contributions

RMF and SMSM carried out the genotyping. RMF carried out the data analysis and drafted the manuscript. BK was responsible for sample recruitment and collection and measurements of anthropometric phenotypes. MNW and BS were responsible for database management. ATH and TMF conceived and designed the study. TMF co-ordinated the study and supervised the redrafting of the manuscript. All authors read and approved the final manuscript.
